# Transcriptomic Profiling Reveals Metabolic and Regulatory Pathways in the Desiccation Tolerance of Mungbean (*Vigna radiata* [L.] R. Wilczek)

**DOI:** 10.3389/fpls.2016.01921

**Published:** 2016-12-21

**Authors:** Xiangrong Tian, Sidi Li, Yisong Liu, Xuanming Liu

**Affiliations:** ^1^College of Biology, Hunan UniversityChangsha, China; ^2^Key Laboratory of Plant Resource Conservation and Utilization of Hunan Province, Jishou UniversityJishou, China; ^3^Center of Analytical Service, Hunan Agricultural UniversityChangsha, China

**Keywords:** mungbean (*Vigna radiate* L. Wilczek), desiccation tolerance, RNA-seq, seeds, transcriptome

## Abstract

Mungbean (*Vigna radiate* L. Wilczek) is an important legume crop for its valuable nutritional and health benefits. Desiccation tolerance (DT) is a capacity of seeds to survive and maintain physiological activities during storage and under stress conditions. Many studies of DT have been reported in other legume crop, such as soybean and *Medicago truncatula* with little studies in the mungbean. In this study, the transcript profiles of mungbean seeds under different imbibition times were investigated for DT using RNA-sequencing (RNA-seq). A total of 3210 differentially expressed genes (DEGs) were found at the key period of DT (3–18 h of imbibition). Gene ontology (GO) and KEGG analysis showed that the terms of “response to stimulus,” “transcription regulator,” “methylation,” and “starch and sucrose metabolism” were enriched for DT. Clustering analysis also showed that many transcription factors (MYB, AP2, and NAC), HSPs, embryogenesis abundant (LEA) proteins, and genes encoding methyltransferase and histone were differentially expressed. Nine of these DEGs were further validated by quantitative RT-PCR (qRT-PCR). Our study extends our knowledge of mungbean transcriptomes and further provides insight into the molecular mechanism of DT as well as new strategies for developing drought-tolerant crops.

## Introduction

Mungbean (*Vigna radiate* L.), a fast-growing legume species, is mainly cultivated in Asia, and also in Australia and Canada (Kang et al., 2014). Mungbean seeds provide a good source of dietary protein and contain higher levels of folate and iron than most other legumes (Keatinge et al., 2011). Mungbean can be processed into flour, soups, porridge and ice cream, making it highly versatile for the human diet, while the forage is beneficial to sheep (Kim et al., 2015). Due to the valuable nutritional and health benefits, great interest in genetic and genomic analyses of the mungbean was aroused for people in developing countries.

Recently, the complete genome sequences of mungbean have been sequenced and accelerate the phenotypes screening for researchers and breeders (Kang et al., 2014). However, not much progress has been performed in downstream analysis using the mungbean genome sequences (Kim et al., 2015). With the advent of next-generation sequencing, RNA-sequencing (RNA-seq) has been used for annotation, gene discovery, and to provide an abundance of genomic resources, including EST-SSR markers (Chen et al., 2015). High-throughput RNA-Seq has been developed for the analysis of transcriptome with or without genomic information. It's an efficient tool to promise simultaneous estimation of transcript abundance and new transcript discovery (Cloonan et al., 2008).

Desiccation tolerance (DT) is defined as the capacity of seeds to survive and maintain their physiological activities during storage and under stress conditions. Orthodox seeds gradually acquire DT during seed maturation (Hoekstra et al., 2001). After undergoing a maturation drying phase, the seeds enter a state of quiescence with metabolically inactive and can be stored for a long time. Once these seeds germinate, tolerance to desiccation is rapidly lost after only a few hours of germination (Vertucci and Farrant, 1995). DT is characteristic of many plants, including *Arabidopsis thaliana* (Maia et al., 2014), maize (Huang et al., 2012), soybean (Blackman et al., 1992), *Medicago truncatula* (Verdier et al., 2013), and *Myrothamnus flabellifoliaus* (Ma et al., 2015). Soybean seeds can be tolerant of dehydration at 6 h of imbibition, but were susceptible to dehydration injury at 36 h of imbition (Senaratna and Mckersie, 1983). Soybean seeds that imbibed for 12–18 h, pea seeds imbibed for 18–24 h and maize seeds imbibed for 48 h were showed a loss of DT (Koster and Leopold, 1988). Previous studies suggested that the accumulation of late embryogenesis abundant (LEA) proteins, soluble sugars, dehydrin antioxidant, and repair system as well as intracellular de-differentiation contribute to DT (Blackman et al., 1992; McDonald, 1999; Pammenter and Berjak, 1999).

Lately, the mechanism of DT in seeds has been studied using transcriptomic and proteomics analysis. In maize, a comparison of the proteome profiles of desiccation-tolerant and desiccation-sensitive maize seeds indicated that 11 proteins were involved in DT (Huang et al., 2012). Co-expression network of developing soybean embryos also revealed some DT associated hubs, including peroxin 19, PATATIN-like protein 6, redox-related GST PH9 protein (Aghamirzaie et al., 2015). Global gene expression analysis of the re-establishment of DT in germinated *A. thaliana* seeds by ABA showed that a set of stress-responsive genes were induced, promoting amplification of signals, growth arrest, protection systems (such as LEA proteins) and adaptation to stress conditions (Costa et al., 2015). Transcriptomic analysis of the resurrection plant *M. flabellifoliaus* revealed approximately 295 transcription factors (TFs) and 484 protein kinases (PKs) were up- or down-regulated in response to desiccation stress (Ma et al., 2015).

As a legume species, mungbean also has a characteristic of DT. However, the molecular mechanism of mungbean seed DT is still largely unknown (Kim et al., 2015). In this study, we attempt to investigate the transcriptome dynamics of mungbean seeds in response to dehydration using RNA-seq. Thus, understanding the extreme tolerance of desiccation and drought in mungbean will aid the development of strategies for improving drought stress resistance in crops.

## Materials and methods

### Plant materials and treatment

The mungbean variety “Zhonglu 1” was used in this study. Mungbean seeds were washed three times with distilled water, and then placed in 12-cm Petri dishes and imbibed in distilled water at 20°C in a dark biochemical incubator. Seeds from each imbibition time were dried silica gel at different dehydration times (5–50 h). Fifty seeds were placed in one plate and three biological replicates were prepared for each time-point sample. Then all the seeds were used for further seed germination and the germination rates and water contents were detected during the dehydration. For RNA-seq, after 3, 6, 18, and 24 h of imbibition, the mungbean seeds were dried for 24 h and collected for sequencing with three replicates for each time-point sample. In order to compare the differentially expressed genes (DEGs) during the imbibition, a sample of each stage without dehydration was used as a control (“ck”), while the seeds with dehydration treatment were used as experiment groups (“sy”). The seeds were collected and immediately frozen in liquid nitrogen, then stored at −80°C until further use.

### RNA extraction, library construction, and sequencing

Total RNA was extracted from the seeds using an RNeasy Plant Mini Kit (QIAGEN) following the manufacturer's protocol. The extracted RNA was treated with DNase I (New England BioLabs) to remove the contaminated DNA. The concentration and quality of each sample was detected by Nanodrop2000. Using Oligo (dT), mRNAs were enriched from total RNA and were randomly cleaved into short fragments. The cDNAs were synthesized using random hexamer primers, RNase H and DNA polymerase I. The double-stand cDNAs were purified and ligated to adaptors for paired-end sequencing. The quality and quantity of the libraries were detected using an Agilent 2100 Bioanalyzer and ABI real time RT-PCR equipment. The qualified cDNA libraries were sequenced by Biomarker Technologies (Beijing, China) using an Illumina HiSeq 2500 platform with PE125.

### Sequence data analysis and annotation

Raw reads were checked with FastQC package (http://www.bioinformatics.bbsrc.ac.uk/projects/fastqc/), and adaptor sequences and low quality reads were removed. A stringent filtering criterion was used to minimize the effects of sequencing errors during assembly. The reads 70% of the bases in a read having high phred quality scores (≥20) were used for assembly and reads lengths shorter than 50 bp were discarded. The obtained clean reads of all twenty four samples were assembled by Trinity v2.1.0 (Released on Sep 29, 2015) with a paired-end model (Grabherr et al., 2011). To annotate the assembled unigenes, sequences were aligned by BLASTx (*E*-value <1e^−5^) to protein databases, including the non-redundant protein (NR) database, Clusters of eukaryotic Orthologous Groups of proteins (COG) database, Swiss-Prot and Kyoto Encyclopedia of Genes and Genomes (KEGG) pathway database. The transcript abundance was normalized by the Fragments Per Kilobase of transcript per Million mapped reads (FPKM) value.

### Analysis of differentially expressed genes

The clean reads of each sample were mapped back to assembled contigs using bowtie2 (Langmead and Salzberg, 2012). The assembled contigs with more than 10 mapped reads were subjected to differential expression analysis. The expression difference of each transcript between different samples was calculated based on a MARS (MA-plot-based method with Random Sampling model) model using the DEGseq package. FDR (false discovery rate) values less than 0.01 and |log2(fold change)|≥2 were considered as significant differences at expression level. To understand the dynamics changes during the different imbibition stages, hierarchical cluster analysis of expression patterns were performed by MultiExpreriment Viewer (v4.1) (Eisen et al., 1998). Using the STRING protein-protein interaction database, we can construct the network with some of the DEGs and TFs. The interaction networks were then visualizd by the software Cytoscape (Shannon et al., 2003).

### GO and KEGG enrichment analysis

Functional annotation of the differential unigenes were performed to search against the NR, Swiss-Prot, GO (Gene Ontology), and KEGG databases. WEGO software was used for functional classification of the results of GO annotations. GO enrichment of DEGs was carried out by ArigGO (Du et al., 2010). GO functional enrichment and KEGG pathway enrichment analysis were also tested at a significance cutoff of *p*-value. All the *p*-values were adjusted with the criterion of Bonferroni correction. We selected the corrected *p*-value of 0.05 as the threshold to determine significant enrichment terms of the gene sets.

### Validation of RNA-seq using quantitative RT-PCR

For the quantitative RT-PCR of the DEGs, 2 μg of total RNA was used to synthesize the cDNA using the RevertAid First Strand cDNA Synthesis Kit (Fermentas). Quantitative RT- PCR was performed using the FastStart Universal SYBR Green Master (Roche) according to the manufacturer's instructions on the StepOne plus Real time PCR Platform (Applied Biosystems). The qRT-PCRs were carried out with the following protocol: 95°C for 10 min, followed by 40 cycles of 95°C for 15 s, and at 60°C for 60 s. The *VgActin* was used as the internal control, which has been shown to be one of the reference genes in mungbean. After amplification, the melting curve was determined for each specific product. Three independent biological replicates for each sample and three technical replicates for each biological replicate were analyzed. All the primers used were listed in Table S1. Significant differences in the expression level between control (ck) and experimantal groups (sy) were evaluated using Student's *t*-test.

### Availability of supporting data

All the sequencing data have been deposited in the NCBI short read archive (SRA) database under the accession number: SRP077637.

## Results

### Desiccation tolerance of mungbean seeds during germination

**Figure 1:**
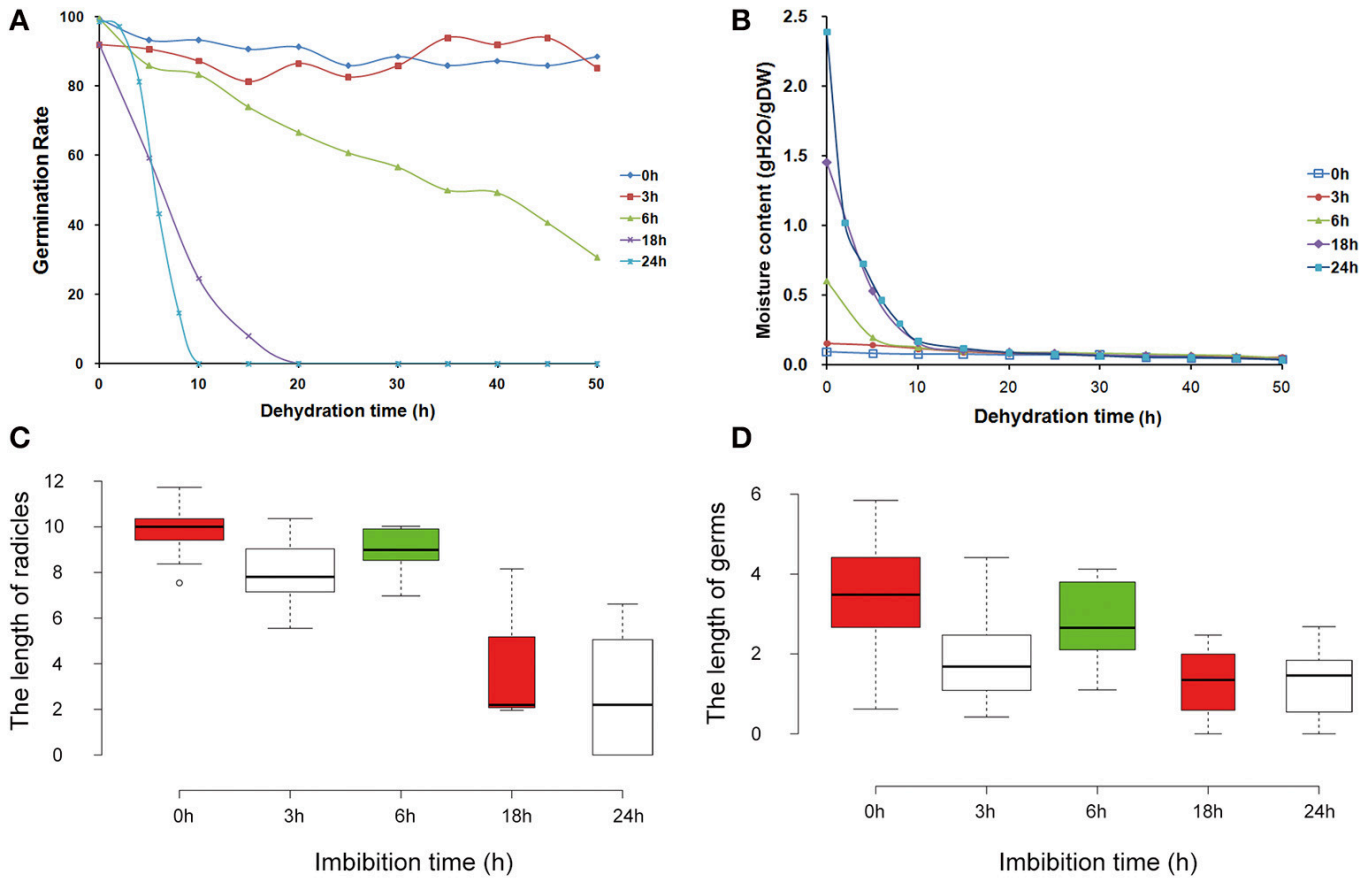
**Morphological and physiological changes of mungbean seeds during the pre-imbibition times (0, 3, 6, 18, and 24 h). (A)** Germination rate of mungbean seeds under different dehydration times. **(B)** Water content of mungbean seeds under different dehydration times. **(C)** The length of radicles under different pre-imbibed times. **(D)** The length of germs under different pre-imbibition times. All the value was average value of three replicates.

In many plants, seeds provide a convenient system for DT, even at the early stages of germination. With increasing imbibition time, the DT was gradually lost. In order to investigate the gene expression associated with DT, different imbibition times were characterized (Figure 1). For mungbean seeds, we observed that the germination rate after 3 h of imbibition was similar to that of seeds without imbibition (Figure 1A), showing 100% during all dehydration times. Germination rate of seeds after 6 h imbibition gradually decreased with increasing dehyration time, while the germination rates were sharply reduced for seeds after 18 and 24 h of imbibition (Figure 1A). In contrast, the moisture contents were high in seeds of the 18 and 24 h of imbibition (Figure 1B). Comparison of the radicles and germs, revealed that the lengths of 0, 3, and 6 h of imbibition had little difference, with significantly decreased in the seeds of 18 and 24 h imbibition (Figures 1C,D). These results suggested that the 3–18 h of imbibition were the key period for DT in mungbean. Therefore, studies on the expression patterns of these imbibition times will reveal the molecular mechanism of DT in mungbean.

### Transcriptome sequencing, assembly, and annotation

To obtain a comprehensive transcriptome profile of DT, 24 libraries of mungbean seeds were constructed with or without dehydration from different time points. Totally, 128.7 Gb clean reads with an average of 4.03 Gb for each sample (Table S2). Using the de novo assembly program Trinity, the contig sequences were created. As a result, a total of 2,544,450 contigs from the 24 samples were assembled (Table 1). And then a total of 420, 228 transcripts and 92,155 unigenes were found. The assembled unigenes had a length distribution from 201 to 39,104 with an average length of 956.02 bp (Figure S1 and Table 1). For each time-point sample, the three biological replicates, showed a high correlation with each other (Figure S2). These assembled unigenes in this study will provide useful resources for further studies in mungbean.

**Table 1:** **The length distribution of assembled contigs, transcripts, and unigenes**.

**Length range**	**Contigs**	**Transcripts**	**Unigenes**
200–300	2,419,095 (95.07%)	0 (0%)	0 (0%)
300–500	55,568 (0.94%)	59,199 (14.09%)	41,883 (45.45%)
500–1000	39,529 (0.56%)	65,304 (15.54%)	26,492 (28.75%)
1000–2000	20,160 (0.26%)	102,735 (24. 45%)	13,410 (14.55%)
>2000	10,096 (0.09%)	192,986 (9.36%)	10,368 (3.21%)
Total number	2,544,450	420,228	92,155
Total length	289,157,880	910,659,696	88,102,372
N50 length	197	3102	1454
Mean length	113.64	2167.06	956.02

For annotation, a similarity search of all unigenes were performed against protein sequences available at NCBI-NR, Swiss-Prot, Pfam and COG databases using BLASTx algorithms with an *E*-value threshold of 1e^−5^ (Table 2). As a result, a total of 62, 811 sequences were annotated, of them, 55,222 (83.90%), 32,041 (51.01%), 45,140 (71.87%), and 30,763 (48.98%) unigenes were aligned against the three protein databases, respectively. GO and KEGG analysis were also performed, generating 32,855 and 18,924 unigenes, respectively (Table 2). The length of these annotated unigenes was mainly ranged from 300 to 1000 bp (65.51%) (Table 2).

**Table 2:** **Annotation of Unigenes were searched against from COG, GO, KEGG, Pfam, SwissProt, NR databases by BLAST**.

**Annotated databases**	**All sequence**	**≥300 bp**	**≥1000 bp**
COG	30,763	20,981	9782
Pfam	45,140	27,314	17,826
Swiss-Prot	32,041	17,875	14,166
NR	55,222	34,306	20,916
GO	32,855	19,717	13,138
KEGG	18,924	11,345	7579
All	62,811	41,167	21,644

### Expression analysis and identification of differentially expressed genes

**Figure 2:**
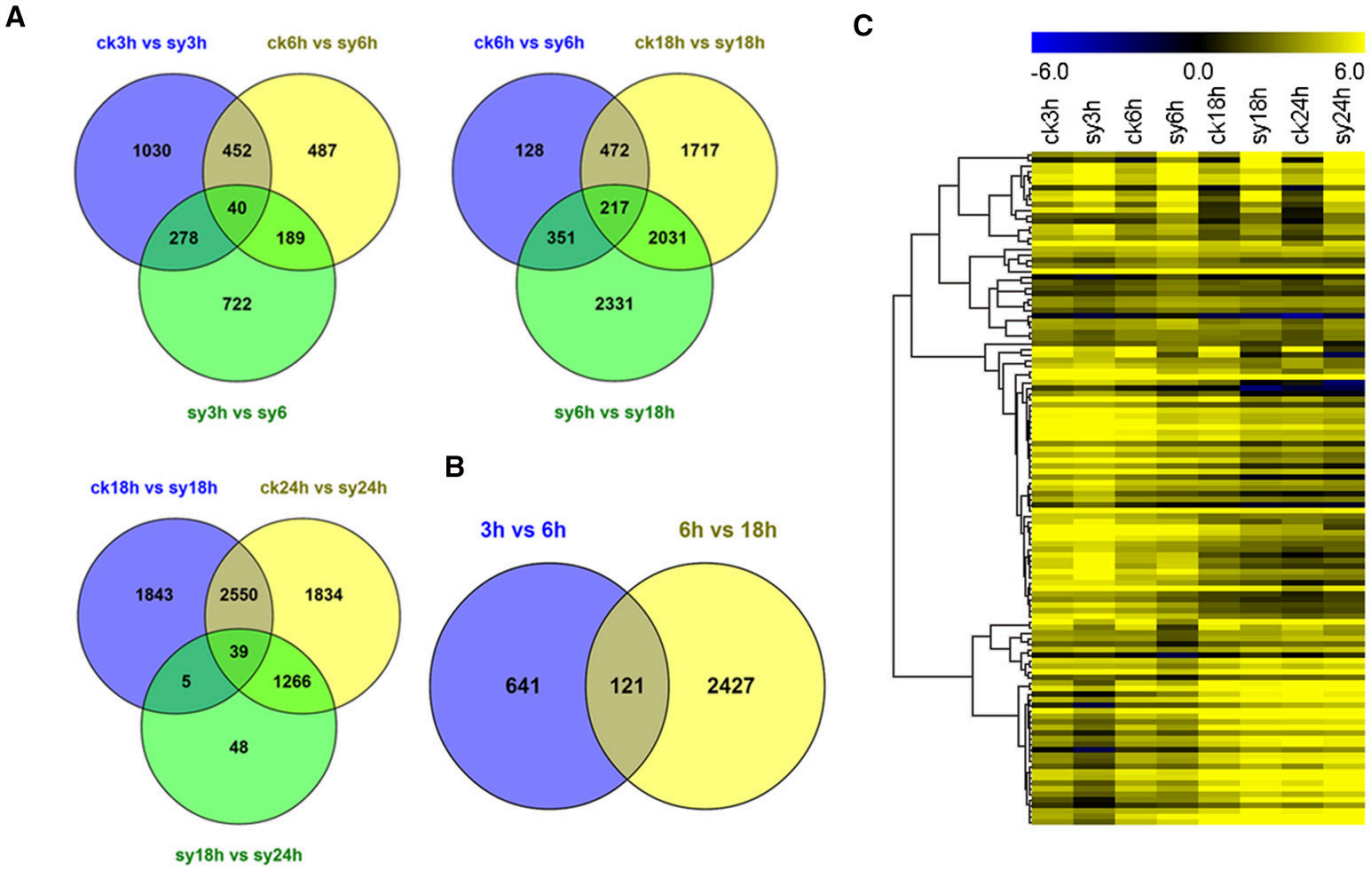
**Comparative analysis of differentially expressed genes (DEGs) among the different imbibition stages. (A)** Venn diagram showing the overlap of DEGs at 3, 6, 18, and 24 h without (“ck”) or with (“sy”) dehydration for DEGs of 3 vs. 6 h, 6 vs. 18 h, and 18 vs. 24 h, respectively. **(B)** Venn diagram showing the overlap of DEGs between 3 vs. 6 h and 6 vs. 18 h. **(C)** Heatmap of 121 shared DEGs expression level at different imbibition stages. All the value at each time point was average value of three replicates.

**Figure 3:**
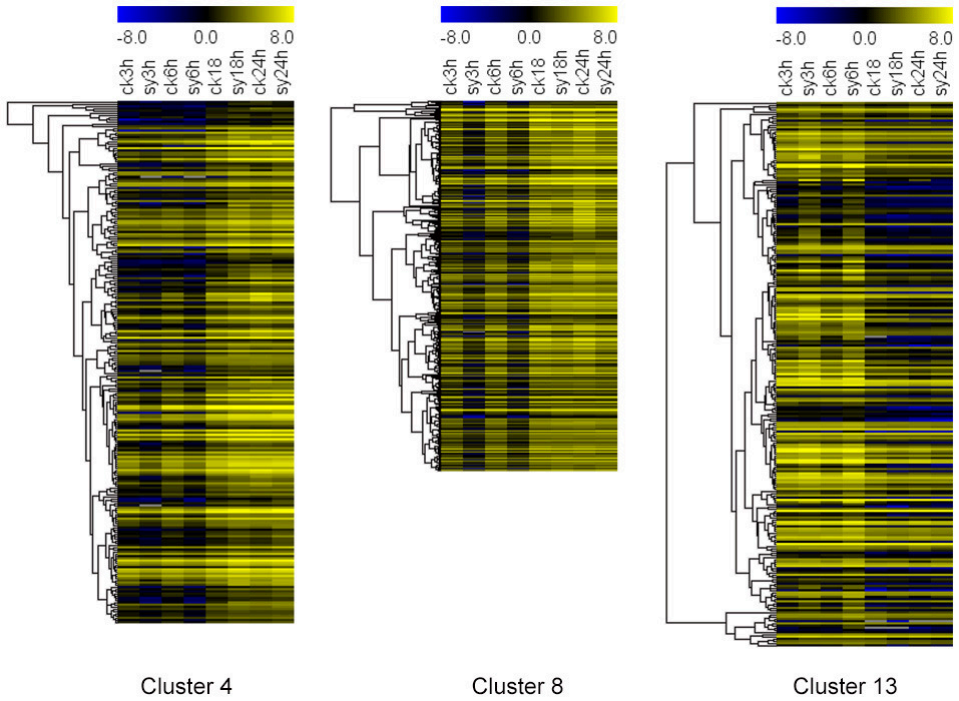
**Heatmaps of three different clusters (cluster 4, 8, and 13) for differentially expressed genes (DEGs) among the 3, 6, and 18 h of imbibition in mungbean seeds**. All the value at each time point was average value of three replicates.

To investigate the gene expression patterns of seeds during the different stages of imbibition, FPKM values were used to normalize the reads from RNA-seq. Thus, the comparison of gene expression patterns between the control and experimental groups or among the experiment groups were performed (Figure 2A). Therefore, the numbers of shared DEGs in mungbean seeds between the adjacent stages after imbibition were 40,217 and 39 for 3 vs. 6 h, 6 vs. 18 h, and 18 vs. 24 h, respectively (Figure 2A). Since 3–18 h imbibition was the key peirod for mungbean seeds DT, the DEGs among these stages were further for in-depth analysis. A total of 121 DEGs were shared at the 3 vs. 6 h and 6 vs. 18 h of imbibition (Figure 2B). A heatmap of the shared DEGs were also showed the difference of their expression patterns, with significant divergence for 3, 6, 18, and 24 h of imbibition (Figure 2C). Clustering analysis of the total DEGs among 3, 6, and 18 h (3310 DEGs) revealed 12 different clusters with differentially expression patterns (Figure S3 and Table S3). Clustes 6, 11, and 13 showed highly expressed DEGs in the experimental groups. Particularly, most DEGs of the cluster 13 were induced at 3 and 6 h of imbibition (Figure 3 and Figure S3). While, other DEGs were particularly down-regulated at 3 and 6 h of imbibition (cluster 4 and cluster 8), suggesting that these DEGs might involved in DT of mungbean seeds (Figure 3).

**Figure 4:**
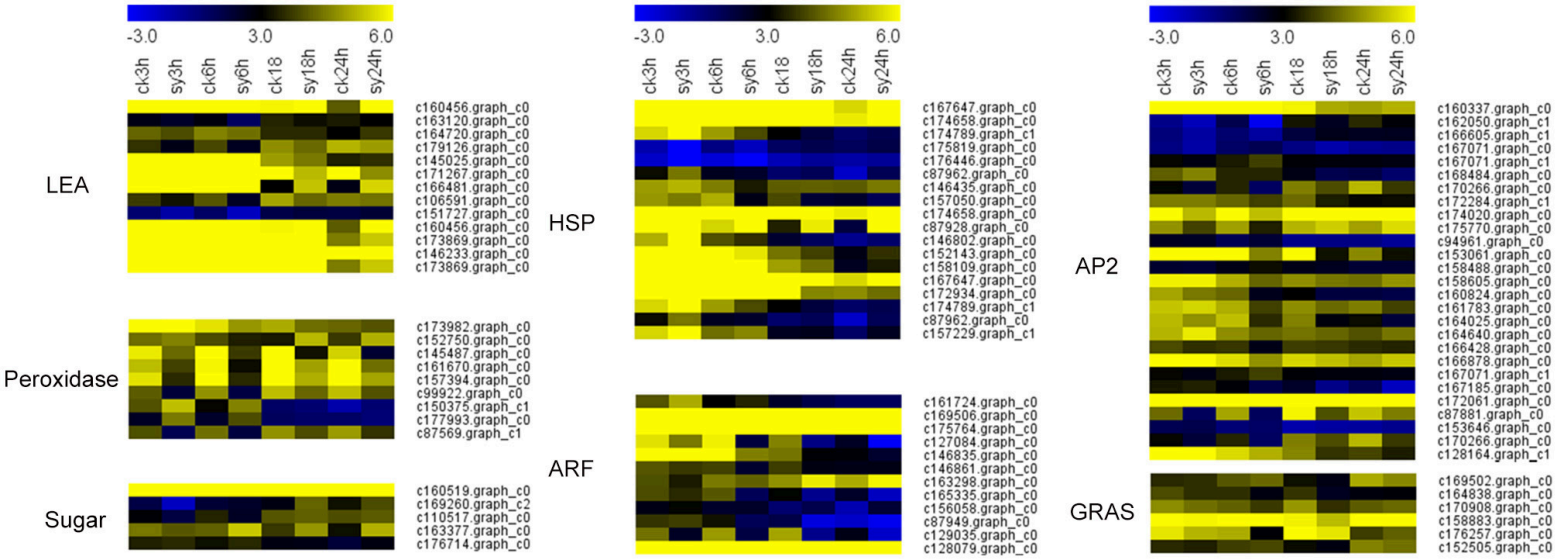
**Heatmaps of differentially expressed genes (DEGs) of mungbean seeds during different imbibition**. LEA, late embryogenesis abundant protein; Sugar, sugar (efflux) transporter; HSP, heat shock protein; ARF, auxin response factor. All the value at each time point was average value of three replicates.

Previous studies have reported that accumulation of protective molecules, including LEA proteins, maturation proteins and antioxidant systems contribute to DT (Maia et al., 2011; Huang et al., 2012). In this study, most of the *LEA* genes (c145025.graph_c0 and c166481.graph_c0) were up-regulated after 3 or 6 h of imbibition (sy3h and sy6h) (Figure 4, **Table 4**, and Table S4). And some of the peroxidases and multicopper oxidases showed differentially expression during the dehydration (Figure 4 and Table S4). Another important component of DT may be the accumulation of a high level of soluble sugars, which were implicated by correlation as adaptive agents for DT during seed development and germination (Blackman et al., 1992). In the present study, sugar transporters (c163377.graph_c0 and c176714.graph_c0) were up-regulated at 6 h of imbibition (Figure 4 and Table S4). Interestingly, some of the DEGs encoding methyltransferases were showed differentially expression at 3 or 6 h of imbibition (**Table 4**, Figure S4 and Table S4). And the expression levels of these DEGs were reduced at the stages (sy3h and sy6h), indicating that methylation invovled in the regulation of DT in mungbean (Figure S4).

### Differential expression of transcription factors during the dehydration

Transcription factors (TFs) play crucial roles in the regulation of gene expression and plant stress responses. In the present study, we also found that many TFs, including HSP, ARF, AP2, MYB, GRAS, WRKY, and NAC, were differentially expressed during the dehydration (Figure 4, **Table 4**, and Table S4). Totally, 14 of the 18 HSP TFs were up-regulated after 3 h of imbibition (Figure 4 and Table S4). Many of the AP2 or ARF TFs, which have important functions in the transcriptional regulation of growth and development as well as stress response, were also differentially expressed during the dehydration process (Figure 4).

### Functional annotation of the differentially expressed genes

**Figure 5:**
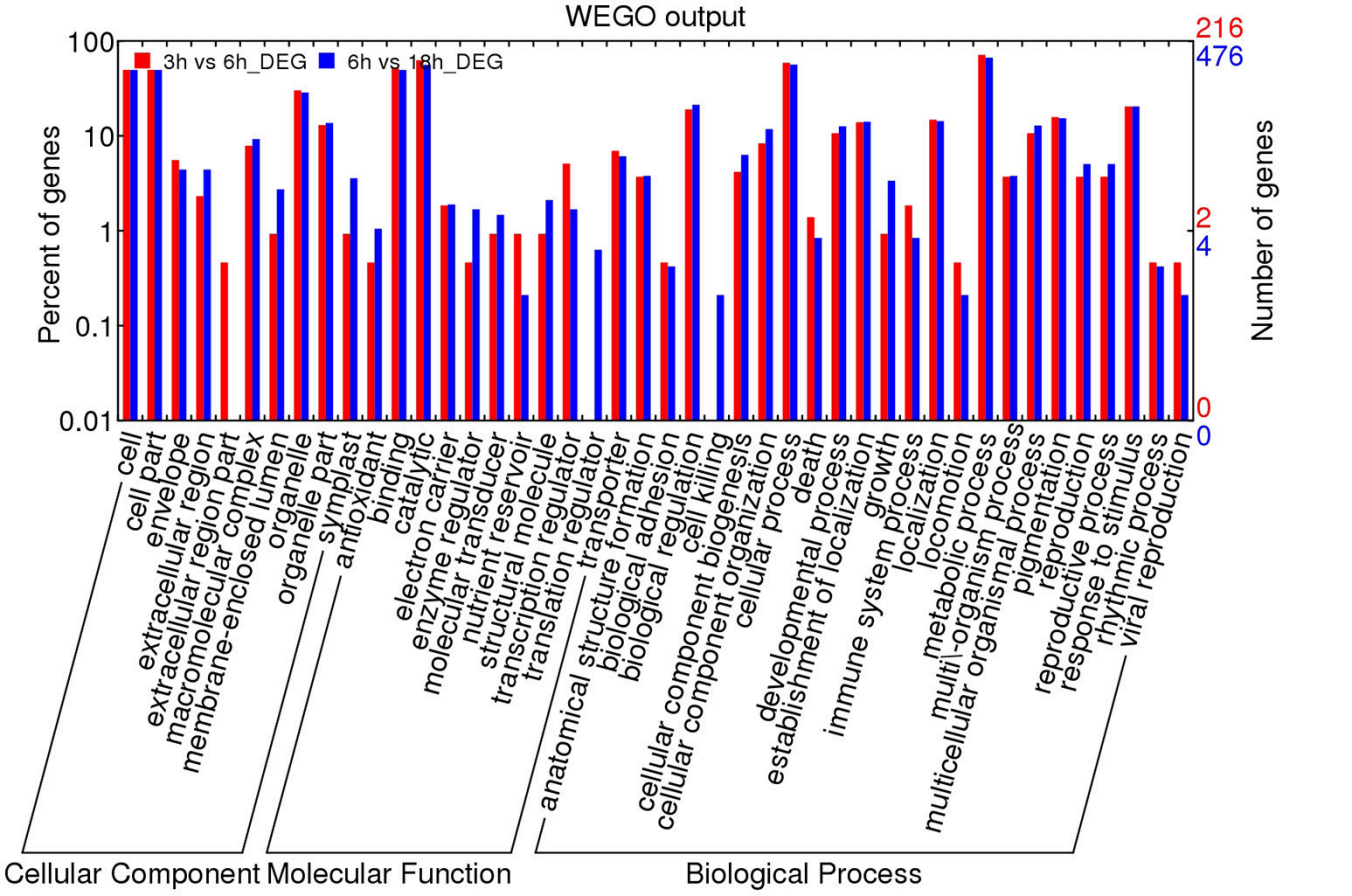
**GO analysis of differentially expressed genes (DEGs) of 3 vs. 6 h and 6 vs. 18 h imbibition in mungbean seeds**. The results were divided into three main categories: molecular function, biological process and cellular component. The right Y-axis indicates the number of genes in up-regulated and down-regulated categories. The left Y-axis indicates the percentage of a specific category of genes in that main category.

**Figure 6:**
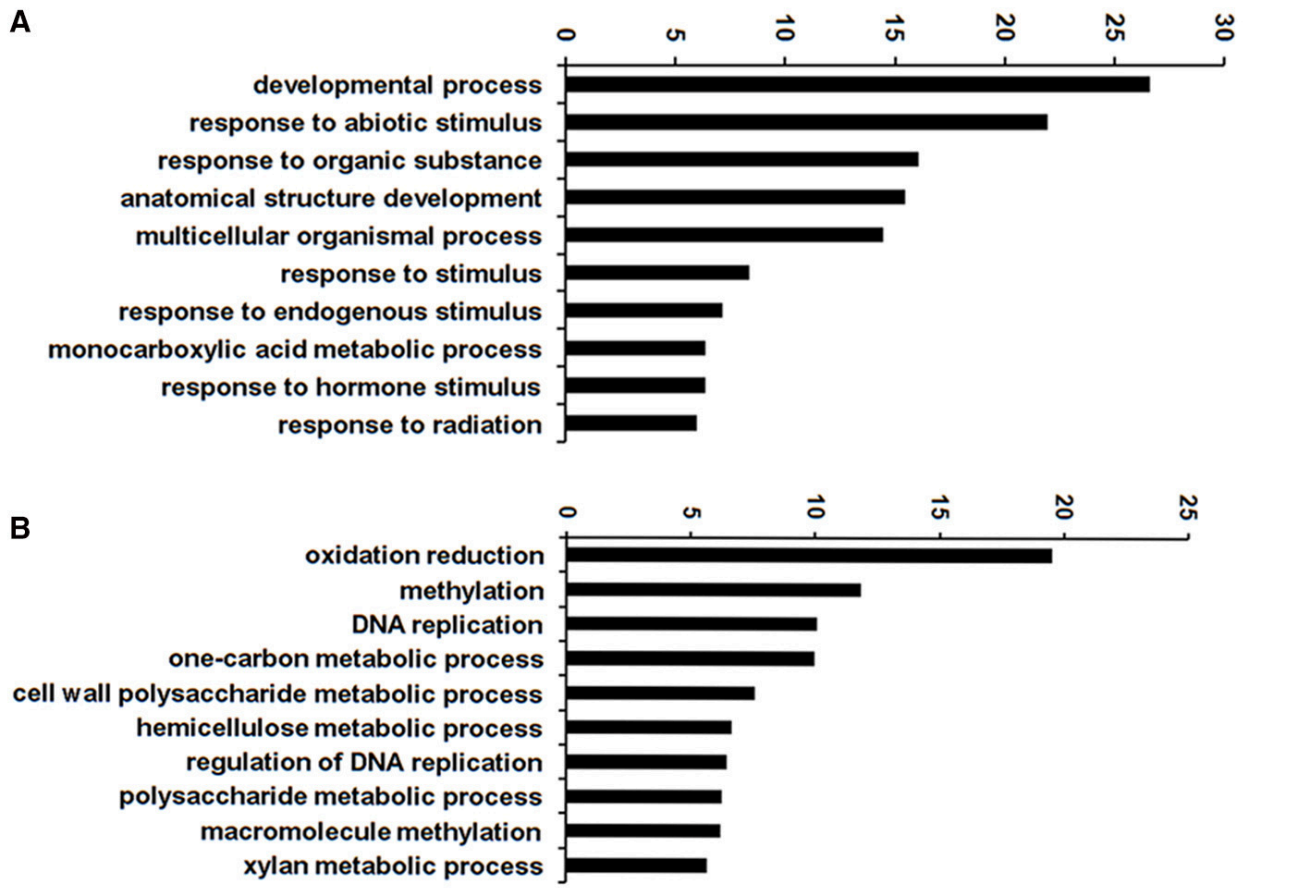
**GO enrichment of differentially expressed genes (DEGs) of 3 vs. 6 h (A)** and 6 vs. 18 h **(B)** imbibition in mungbean seeds. Top 10 enriched GO terms were shown here.

To infer the biological processes and functions of genes associated with DT, we conducted GO analysis of the DEGs at different stages. In total, 32,855 transcripts were assigned to 3458 functional terms and the GO terms of DEGs from the adjacent stages of the key period of DT were enriched (Figures 5, 6 and Table S5). The assigned functions of expressed transcripts covered a broad range of GO categories. Particularly, “extracellular region part” and “transcription regulator” were over-represented in DEGs of 3 vs. 6 h, while “cell killing,” “translation regulator,” and “growth” were the predominant groups for the DEGs at 6 vs. 18 h (Figure 5). GO enrichment analysis also showed that more DEGs were involved in “developmental process” and “response to stimulus” processes during early imbibition (3 vs. 6 h) (Figure 6A). Moreover, for 6 vs. 18 h after imbibition, “oxidation reduction,” “methylation,” and “cell wall polysaccharide metabolism” were the predominant categories (Figure 6B). Thus, the DEGs along with the time of dehydration treatment can be divided into two phases: response to stimulus, and oxidation reduction or metabolic process (Figure 6 and Table S5). The antioxidant systems and membrane structural stabilizing were essential to DT in resurrection plants (Moore et al., 2009).

In order to mine the genes involved in DT, 18,924 non-redundant potential protein sequences were searched against the KEGG database (Table S6). A total of 324 KEGG pathways were identified for the DEGs from the adjacent stages. The top 10 pathways enriched for the DEGs of 3 vs. 6 h and 6 vs. 18 h after imbibition were shown in Table 3. Most pathways were involved in metabolic processes, such as carbon metabolism (ko01200), starch and sucrose metabolism (ko00500) and amino sugar and nucleotide sugar metabolism (ko00520). However, there were some different KEGG pathways between 3 vs. 6 h and 6 vs. 18 h after imbibition. For the DEGs between 3 and 6 h after imbibition, pathways of fatty acid metabolism, pyruvate metabolism, and carbon fixation in photosynthetic organisms were enriched. Whereas, more DEGs were involved in the pathways of 6 vs. 18 h, and phenylpropanoid biosynthesis, phenylalanine metabolism, cysteine and methionine metabolism and ribosome pathways were overrepresented (Table 3). Sucrose was known membrane protectants, stabilizing the cellular processes, while some amino acid can be compatible solutes (Moore et al., 2009). Therefore, the results suggested different pathways were occurred during the loss of DT. Furthermore, COG analysis also showed that DEGs related to “transcription,” “cell wall/membrane,” and “lipid metabolism” were differentially expressed at 3–18 h of imbibition (Figure S5).

**Table 3:** **List of the top 10 pathways of DEGs among the adjacent stages of imbibition in mungbean**.

**KEGG ID**	**Pathway**	**Number of unigenes**
**3 vs. 6 h**		
ko01200	Carbon metabolism	26
ko01230	Biosynthesis of amino acids	25
ko00500	Starch and sucrose metabolism	23
ko04075	Plant hormone signal transduction	22
ko04141	Protein processing in endoplasmic reticulum	21
ko01212	Fatty acid metabolism	15
ko00620	Pyruvate metabolism	14
ko00040	Pentose and glucuronate interconversions	13
ko00710	Carbon fixation in photosynthetic organisms	12
ko00520	Amino sugar and nucleotide sugar metabolism	12
**6 vs. 18 h**		
ko01200	Carbon metabolism	62
ko00500	Starch and sucrose metabolism	53
ko00940	Phenylpropanoid biosynthesis	52
ko04075	Plant hormone signal transduction	52
ko01230	Biosynthesis of amino acids	51
ko00360	Phenylalanine metabolism	34
ko00040	Pentose and glucuronate interconversions	33
ko00270	Cysteine and methionine metabolism	31
ko00520	Amino sugar and nucleotide sugar metabolism	31
ko03010	Ribosome	30

### Validation of RNA-seq data by quantitative RT-PCR

**Figure 7:**
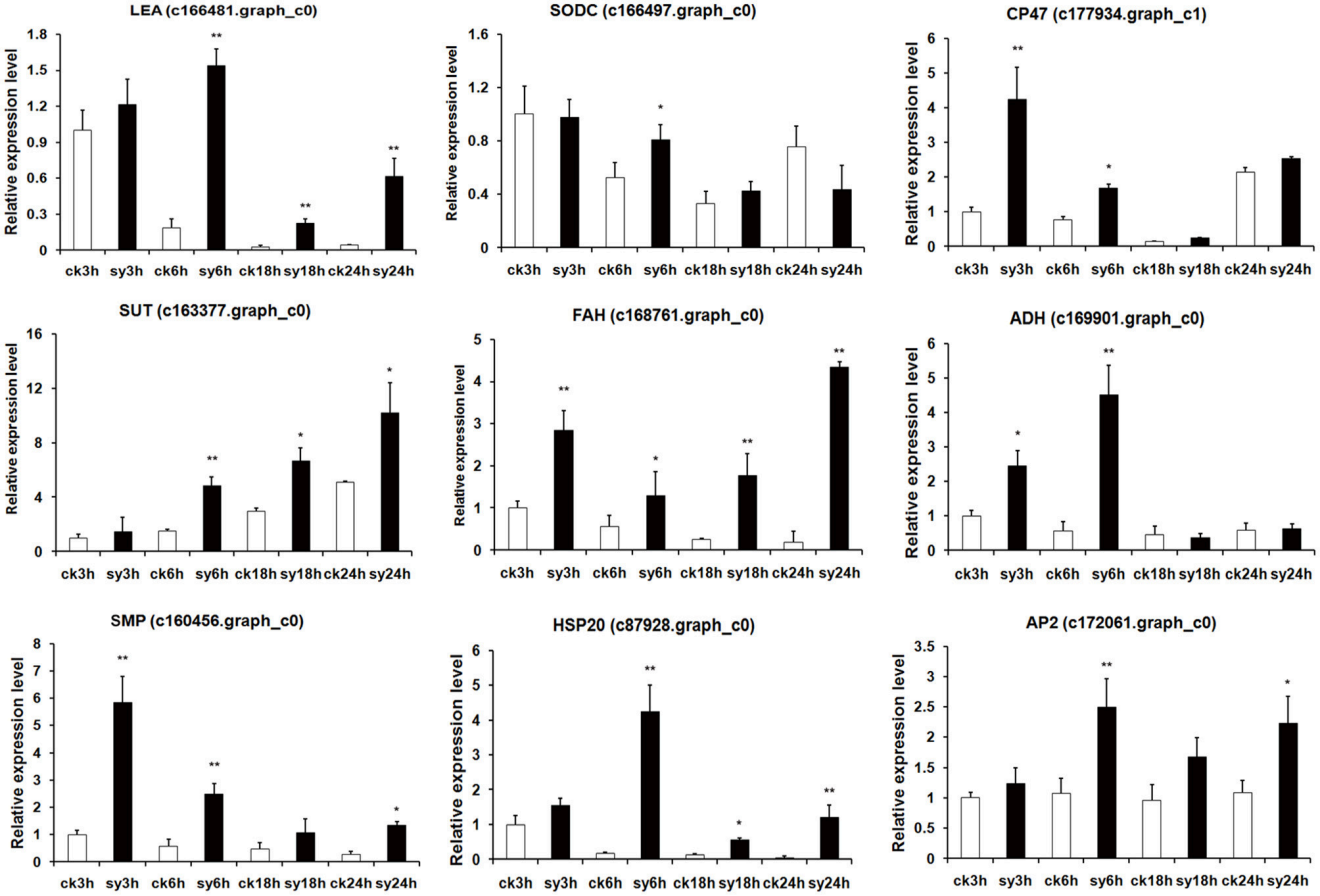
**Quantitative RT-PCR validation of differentially expressed genes (DEGs) of 3 vs. 6 h and 6 vs. 18 h imbibition in mungbean seeds**. LEA, late embryogenesis abundant protein; SODC, copper/zinc superoxide dismutase; CP47, photosystem II CP47 reaction center protein; SUT, sugar efflux transporter; FAH, fatty acid hydroxylase; ADH, aldehyde dehydrogenase; SMP, seed maturation protein; HSP20, heat shock 20 kDa protein; AP2, AP2-like transcription factor. The asterisks indicate significant differences between “sy” and “ck” as determined by Student's *t*-test (^*^P < 0.05, ^**^P < 0.01).

**Figure 8:**
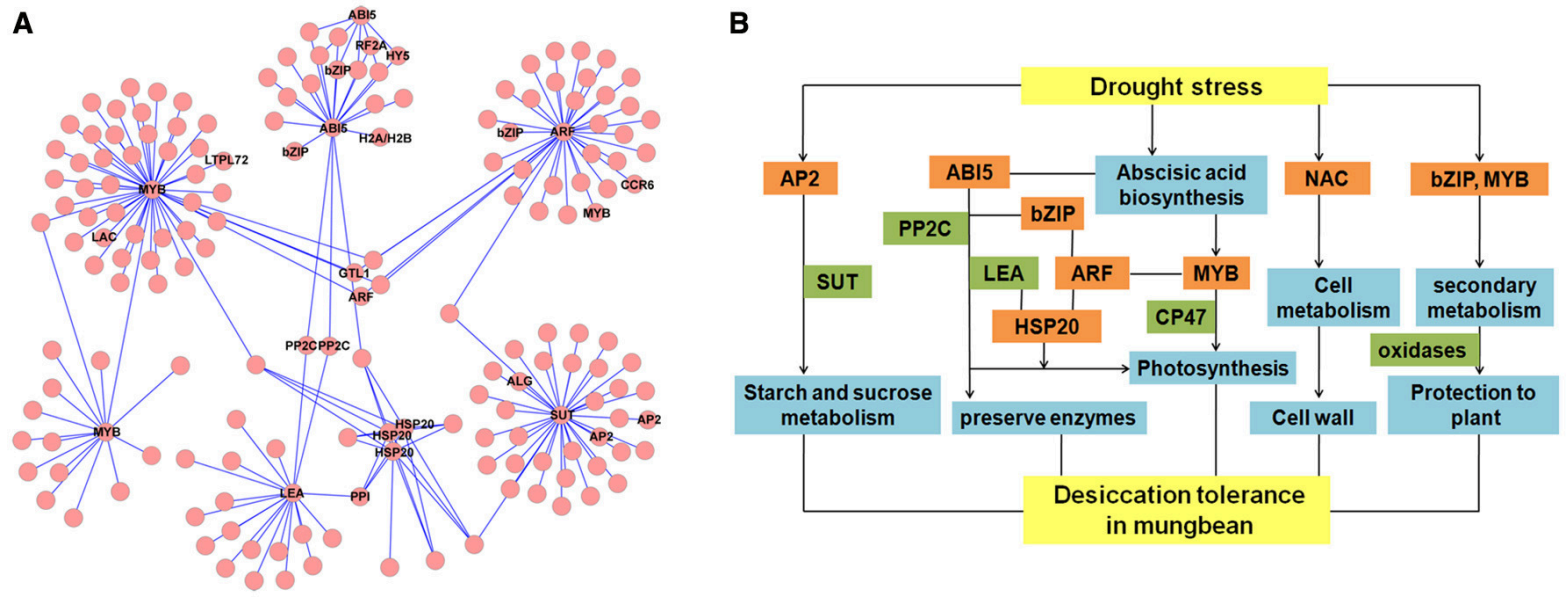
**Regulatory network and hypothetical network model of desiccation tolerance (DT) in mungbean. (A)** Network of differentially expressed genes (DEGs) involved in DT of mungbean seeds. LEA, late embryogenesis abundant protein (c166481.graph_c0); SUT, sugar transporter (c163377.graph_c0); HSP20, heat shock 20 kDa protein (c87928.graph_c0,c87962.graph_c0, c174658.graph_c0,); AP2, AP2-like transcription factor (c172061.graph_c0, c94961.graph _c0); ABI5, ABSCISIC ACID-INSENSITIVE 5 (c192685.graph_c0); ARF, auxin response factor (c128079.graph_c0). **(B)** Hypothetical model of DT transcriptional network in mungbean.

To assess the reliability of the RNA-seq data, quantitative RT-PCR analysis was performed using gene-specific primers of nine randomly selected genes. As expected, LEA protein (c166481.graph_c0) and seed maturation protein (c160456.graph_c0), which are involved in DT, showed highly expression levels at all the experiment groups. Sugar transporter (SUT, c163377.graph_c0) and copper/zinc superoxide dismutase (SODC, c166497.graph_c0) were induced at sy6h (Figure 7). Interestingly, photosystem II CP47 reaction center protein (c177934.graph_c1) was significantly highly expressed at 3 and 6 h of imbibition. Two enzymes, fatty acid hydroxylase (c168761.graph_c0) and aldehyde dehydrogenase (ADH, c169901.graph_c0) were also validated to have differentially expression patterns during the different stages (Figure 7). In addition, two TFs (HSP20, c87928.graph_c0 and AP2, c172061.graph_c0) were also up-regulated at 6 h of imbibition (Figure 7). The expression profiles of these nine detected genes showed the same trend and consistent results between the qRT-PCR and RNA-seq. Network analysis were also revealed that some of the DEGs were involved in diverse signaling pathways mediated by hormones (auxin and ABA) (Figure 8A). The TFs were closely connected with LEA and SUT, which might suggest a contribution to the regulation of DT in mungbean.

## Discussion

DT is the capacity of seeds to survive and maintain their physiological activities when under a rigorous drying process. In the past few years, desiccation tolerance in the seeds of soybean, Arabidopsis, maize, and *M. truncatula* have been well studied (Blackman et al., 1992; Wu et al., 2009; Maia et al., 2011, 2014; Verdier et al., 2013). However, little studies have been reported on the DT of mungbean seeds. In this study, we combined phenotypic analysis with RNA-Seq to investigate the phenotypic changes and transcriptomic basis of DT in mungbean seeds during germination.

Like the soybean seeds, the viability of mungbean seeds after imbibition-dehydration treatment was affected by the severity of the dehydration treatment and the germination times prior to dehydration (Senaratna and Mckersie, 1983). In mungbean seeds, DT remianed until 6 h of imbibition, after which it was progressively lost, particularly at 18 and 24 h of imbibition (Figure 1). As imbibition progressed, the germination rate of the seeds were decreased when dehydration (Figure 1). This indicated that the mechanisms associated with the maintenance of DT were inactivated with the progress of the germination process, contributing to an increase in the sensitivity of the seeds to desiccation. These results were consistent with previous studies (Bewley and Black, 1994). Therefore, 3–18 h of imbibition was the key period for tolerance to desiccation and further gene expression analysis will reveal the mechanism of DT in mungbean.

A total of 3310 DEGs were identified in mungbean seeds at 3–18 h of imbibition (Figure 2). Clustering analysis showed that many DEGs showed different expression patterns in sy3h and sy6h from that of sy18h and sy24h (Figure 3 and Table S3). GO enrichment analysis revealed that “extracellular region part,” “transcription regulator,” and “response to stimulus” were over-represented at the 3 and 6 h of imbibition (sy3h and sy6h; Figure 6A). These genes included salt stress response/antifungal protein, universal stress protein, HSPs (HSP70, HSP20), and many TFs (MYB, AP2, ARF, WRKY, NAC, and GRAS) (Figures 4, 7 and Tables S3, S4). Moreover, for 6 vs. 18 h after imbibition, “oxidation reduction,” “methylation,” and “cell wall polysaccharide metabolism” were the predominant categories (Figure 6B). For example, the DEGs included copper/zinc superoxide dismutase (SODC), peroxidase, multicopper oxidase, methyltransferase, histone deacetylase complex subunits 2 and core histone H2A/H2B/H3/H4 (Table S4). These results suggested that a number of genes were differentially expressed for immediate response to dehydration. The TFs and histone were also involved in the regulation of DT in mungbean. KEGG pathway analysis showed that carbon metabolism and sugar metabolism were enriched at 3–18 h of imbibition (Table 3 and Table S6), indicating the role of soluble sugars in the DT of mungbean.

It is interesting that some DEGs associated with methylation were identified in the mungbean seeds during dehydration (Figure 6B, Figure S4, and Table S4). Many DEGs encoding methyltransferases were down-regulated in the seeds at 3 and 6 h of imbibition (Table 4 and Table S4). Increases in global DNA methylation are known to inhibit gene expression, while a reduction in methylation enhances gene expression (Zemach et al., 2010; Hu et al., 2015). Thus, the decrease of methylation at 3 and 6 h of imbibition might active the expression of some genes, which will be involved in DT. In common pear seeds during desiccation, a slight decrease in global DNA methylation from 8.2–8.8 to 4.8–5.3% were observed (Michalak et al., 2013). DNA methylation was also affected by desiccation in orthodox seeds of *Acer platanoides* and played a relevant role in DT in seeds (Plitta et al., 2014). Therefore, DNA methylaton observed in mungbean seeds in response to desiccation might have an influence on the gene expression of specific genes for DT.

**Table 4:** **Expression analysis of mungbean genes related to either regulation or protection that up-regulated in the experiment groups at key period for desiccation tolerance**.

**ID**	**Annotation**	**sy3h/ck3h**	**sy6h/ck6h**	**sy18h/ck18h**
**TRANSCRIPTION FACTORS (TF)**				
c128040.graph_c0	Myb-like DNA-binding domain	1.89	3.44	2.29
c175764.graph_c0	Auxin response factor	1.58	2.11	1.11
c149104.graph_c0	NAC transcription factor NAM-B2	1.98	5.85	1.51
c144929.graph_c0	No apical meristem (NAM) protein	2.41	10.04	7.64
c151939.graph_c0	Transcription factor bHLH48	1.29	1.31	1.04
**CELLULAR COMMUNICATION**				
c151932.graph_c0	Zinc finger C-x8-C-x5-C-x3-H type	1.61	2.78	5.72
c171933.graph_c0	Protein kinase	1.70	5.51	9.21
c171575.graph_c0	Protein tyrosine kinase	1.44	2.80	5.44
**HEAT SHOCK PROTEINS**				
c167647.graph_c0	Hsp20/alpha crystallin family	3.01	1.79	1.59
c87928.graph_c0	Hsp20/alpha crystallin family	3.98	6.01	5.31
c165607.graph_c1	HSP70	1.89	3.62	5.46
**LEA PROTEINS**				
c166481.graph_c0	Late embryogenesis abundant (LEA)	3.60	14.99	5.06
c160456.graph_c0	Late embryogenesis abundant protein	2.56	1.64	2.04
**STRESS PROTEINS**				
c173869.graph_c0	Seed maturation protein	2.88	1.82	1.05
c160456.graph_c0	Seed maturation protein	2.56	1.64	2.04
**DETOXIFICATION**				
c158715.graph_c0	Peroxidase	2.75	6.65	7.11
c166497.graph_c0	Copper/zinc superoxide dismutase	1.75	1.17	4.09
**METHYLATION**				
c168324.graph_c0	O-methyltransferase (PCMT)	2.40	2.27	2.42
c173726.graph_c1	Histone deacetylase domain	2.40	1.29	1.43

In the present study, we found many LEA genes were up-regulated at the 3 and 6 h of imbibition (Figures 4, 7, Table 4, and Table S4). LEA proteins are a family of intrinsically disordered proteins which accumulate during dehydration-tolerant stages of development in many plant species (Tunnacliffe and Wise, 2007; Battaglia et al., 2008). LEA proteins have been reported to preserve the activity of some enzymes such as CS (citrate synthase) and LDH (lactate dehydrogenase) after desiccation (Battaglia et al., 2008). Therefore, the accumulation of LEA proteins might preserve the activity of enzymes (CS) and stabilize the membranes in mungbean seeds (Table S4).

The sugar transporter is responsible for sucrose uptake in many plants. In our study, the genes encoding the sugar transporter (for example, c163377.graph_c0) were highly expressed in the experimental groups (Figure 7 and Table S4), which might increase the soluble sugar content in mungbean seeds. Soluble sugars are highly sensitive to environmental stress and are effective membrane protectants. They can replace water in the hydration shell and prevent membrane fusion, allowing the preferential hydration of the membrane surface (Hu et al., 2016). In germinating soybean, sucrose served as the principal agent of DT in the seeds because of sucrose and larger oligosaccharides were consistently present during the tolerance stages (Koster and Leopold, 1988; Blackman et al., 1992). These differentially expressed SUTs also demonstrated that the soluble sugars were involved in the DT of mungbean (Figure 7).

GO and KEGG analyses showed that many DEGs were enriched in “extracellular region part,” “cell wall polysaccharide metabolism,” and pathways of “fatty acid metabolism” (Figure 5 and Table 3). During the initial period of treatment (3 and 6 h), many DEGs response to the stimulus, then oxidation and protective systems were started (6 and 18 h) (Table S5). Dehydration of the seeds after a critical stage perturbs these membrane systems and irreversibly blocks the developmental process, and cannot continue upon reimbibition (Senaratna and Mckersie, 1983). And the lipid composition changes contribute to membrane stabilization, indicating that the maintenance of membrane structure was essential for DT during dehydration (Gasulla et al., 2013). These DEGs were also found to be associated with DT in mungbean. Many ABC transporters were also differentially expressed in the mungbean seeds. Previous studies have reported that the ATP binding cassette transporter, Pp-ABCG7, was required for cuticular wax deposition and DT in the moss *Physcomitrella patens* (Buda et al., 2013). These results suggested that ABC transporters were invovled in cuticular wax accumulation for the DT of mungbean.

During the dehydration of mungbean, lots of HSPs were also differentially expressed in the seeds (Figures 4, 7, Table 4, and Table S4). HSPs are responsible for protein folding, and can assist in protein refolding under stress conditions (Wu et al., 2015; Carniel et al., 2016; Wang et al., 2016). Overexpression of the HaHSFA9 and HaHSFA4a in tobacco demonstrated their roles in DT (Almoguera et al., 2015). Therefore, the HSPs might also invovled in the DT of mungbean. Forthermore, dehydration also affected the gene expression of components of the photosynthetic apparatus ROS-scavenging system and plant hormone signal transduction. In the present study, many of these DEGs were also found, for example, CP47 (c177934.graph_c1), SODC (c166497. graph_c0), ARF (c163298.graph_c0) (Figure 7 and Table S4).

Like other species, many TFs were differentially expressed during the dehydration in mungbean, including MYB, AP2, HSP20, NAC, WRKY, homeodomain-leucinezipper (HD-Zip), basic leucine zipperdomain (bZIP), zinc finger, and GRAS (Figures 7, 8 and Tables S3, S4). In Arabidopsis, overexpressing CpMYB10 was increased the tolerance to drought and salt stress (Villalobos et al., 2004; Moore et al., 2009; Mohanty et al., 2016). In our study, we also found some MYB TFs were invovled in the regulation network (Figure 8A). Furthermore, ABI5, PP2C, AP2/EREPB, and HSP20 TFs were demonstrated to connect with LEA or SUT (Figure 8). The genes (ABI5 and PP2C) were important components of ABA signaling pathway that regulates survival in the dry state (Verdier et al., 2013; González-Morales et al., 2016). Based on the information, we proposed the possible metabolic network of DT in the mungbean (Figure 8B). The two TFs (HSP20 and AP2) differentially expressed during the key period of DT (3–18 h of imbibition) also suggested their regulation roles in mungbean seeds for DT (Figure 7). In Arabidopsis, HSP17.4 has been revealed to protective membranes against desiccation throughout the seed (Wehmeyer and Vierling, 2000). And AP2/EREPB TF (c172061. graph_c0) belonged to the DREB-A2 family which was reported involved in ABA signaling pathway (Verdier et al., 2013). Therefore, further functional charaterizaton of the two TFs and the pathway will shed light on their roles in DT of mungbean.

## Conclusions

Desiccation tolerance is a characteristic of many higher plant seeds and can maintain their physiological activities during storage and under stress conditions. Using RNA-seq, the transcriptome of mungbean seeds during dehydration was analyzed. These results identified 3310 DEGs, including LEA proteins, soluble sugars and HSPs, which might be involved in the DT in mungbean seeds. Furthermore, many TFs and DNA methylation related proteins were involved in the regulation of DT in mungbean. And regulation network analysis suggested that HSP20 and AP2 played important role in DT. More functional analysis of the two TFs would provide critical clues to reveal the molecular mechanisms for DT in mungbean.

## Author contributions

Designed the experiments: XT, XL. Performed the experiments: XT, SL, and YL. Analyzed the data: XT, SL. Wrote the paper: XT, XL. All authors read and approved the final manuscript.

### Conflict of interest statement

The authors declare that the research was conducted in the absence of any commercial or financial relationships that could be construed as a potential conflict of interest.

## Supplementary material

Figure S1 **Distribution of the length of transcript assembly of unigenes**.

Figure S2 **Heatmap of the correlation of the 24 different samples of mungbean**.

Figure S3 **Clustering analysis of the differentially expressed genes (DEGs) at 3–18 h of imbibition**.

Figure S4 **Boxplots of the expression levels of DEGs encoding methyltransferase at different stages in mungbean seeds**.

Figure S5 **COG functional classification of differentially expressed genes (DEGs) at 3–18 h of imbibition. (A)** 3 h, **(B)** 6 h, and **(C)** 18 h.

Table S1 **Primers used in the study for quantitative RT-PCR**.

Table S2 **Statistical analysis of the sequencing reads in 24 libraries that mapped to the mungbean reference genome**.

Table S3 **Cluster analysis of the differentially expressed genes (DEGs) among the different stages of imbibition in mungbean**. From left to right of X aixs were ck3h, sy3h, ck6h, sy6h, ck18h, sy18h, ck24h, and sy24h, respectively.

Table S4 **Some of the differentially expressed genes (DEGs) related to desiccation tolerance in mungbean seeds**.

Table S5 **Gene Ontology enrichments of differentially expressed genes (DEGs) during different stages of imbibition in mungbean seeds**.

Table S6 **KEGG pathway enrichment of differentially expressed genes (DEGs) during different stages of imbibition in mungbean seeds**.

## References

[B1] Aghamirzaie D. Batra D. Heath L. S. Schneider A. Grene R. Collakova E. (2015). Transcriptome wide functional characterization reveals novel relationships among differentially expressed transcripts in developing soybean embryos. BMC Genomics 16:928. 10.1186/s12864-015-2108-x 26572793 PMC4647491

[B2] Almoguera C. Personat J. M. Dapena P. Jordano J. (2015). Heat shock transcription factors involved in seed desiccation tolerance and longevity retard vegetative senescence in transgenic tobacco. Planta 242, 461–475. 10.1007/s00425-015-2336-y 26021607

[B3] Battaglia M. Olvera-Carrillo Y. Garciarrubio A. Campos F. Covarrubias A. A. (2008). The enigmatic LEA proteins and other hydrophilins. Plant Physiol. 148, 6–24. 10.1104/pp.108.120725 18772351 PMC2528095

[B4] Bewley J. D. Black M. (1994). Physiology of Development and Germination, 2nd Edn. New York, NY: Plenum Press.

[B5] Blackman S. A. Obendorf R. L. Leopold A. C. (1992). Maturation proteins and sugars in desiccation tolerance of developing soybean seeds. Plant Physiol. 100, 225–230. 10.1104/pp.100.1.225 16652951 PMC1075542

[B6] Buda G. J. Barnes W. J. Fich E. A. Park S. Yeats T. H. Zhao L. X.. (2013). An ATP binding cassette transporter is required for cuticular wax deposition and desiccation tolerance in the moss *Physcomitrella patens*. Plant Cell 25, 4000–4013. 10.1105/tpc.113.117648 24163310 PMC3877811

[B7] Carniel F. C. Gerdol M. Montagner A. Banchi E. Moro G. D. Manfrin C.. (2016). New features of desiccation tolerance in the lichen photobiont Trebouxia gelatinosa are revealed by a transcriptome approach. Plant Mol. Biol. 91, 319–339. 10.1007/s11103-016-0468-5 26992400

[B8] Chen H. Lu C. Jiang H. Peng J. (2015). Transcriptome analysis reveals distinct Aluminum-tolerance pathways in the Al-accumulating species Hydrangea macrophylla and Marker identification. PLoS ONE 10:e0144927. 10.1371/journal.pone.0144927 26660093 PMC4682798

[B9] Cloonan N. Forrest A. R. Kolle G. Gardiner B. B. Faulkner G. J. Brown M. K.. (2008). Stem cell transcriptome profiling via massive-scale mRNA sequencing. Nat. Methods 5, 613–619. 10.1038/nmeth.1223 18516046

[B10] Costa M. C. Righetti K. Nijveen H. Yazdanpanah F. Ligterink W. Buitink J.. (2015). A gene co-expression network predicts functional genes controlling the re-establishment of desiccation tolerance in germinated *Arabidopsis thaliana* seeds. Planta 242, 435–449. 10.1007/s00425-015-2283-7 25809152 PMC4498281

[B11] Du Z. Zhou X. Ling Y. Zhang Z. H. Su Z. (2010). agriGO: a GO analysis toolkit for the agricultural community. Nucleic Acid Res. 38, W64–W70. 10.1093/nar/gkq310 20435677 PMC2896167

[B12] Eisen M. B. Spellman P. T. Brown P. O. Botstein D. (1998). Cluster analysis and display of genome-wide expression patterns. Proc. Natl. Acad. Sci. U.S.A. 95, 14863–14868. 10.1073/pnas.95.25.14863 9843981 PMC24541

[B13] Gasulla F. Dorp K. V. Dombrink I. Zähringer U. Gisch N. Dormann P.. (2013). The role of lipid metabolism in the acquisition of desiccation tolerance in Craterostigma plantagineum: a comparative approach. Plant J. 75, 726–741. 10.1111/tpj.12241 23672245

[B14] González-Morales S. I. Chávez-Montes R. A. Hayano-Kanashiro C. Alejo-Jacuinde G. A. Rico-Cambron T. Y. de Folter S. (2016). Regulatory network analysis reveals novel regulators of seed desiccation tolerance in *Arabidopsis thaliana*. Proc. Natl. Acad. Sci. U.S.A. 8, E5232–E5241. 10.1073/pnas.1610985113 PMC5024642 27551092

[B15] Grabherr M. G. Haas B. G. Yassour M. Levin J. Z. Thompson D. A. Amit I.. (2011). Full-length transcriptome assembly from RNA-Seq data without a reference genome. Nat. Biotechnol. 29, 644–652. 10.1038/nbt.1883 21572440 PMC3571712

[B16] Hoekstra F. A. Golovina E. A. Buitink J. (2001). Mechanisms of plant desiccation tolerance. Trends Plant Sci. 9, 431–438. 10.1016/S1360-1385(01)02052-0 11544133

[B17] Hu J. Chen X. Zhang H. Ding Y. (2015). Genome-wide analysis of DNA methylation in photoperiod- and thermo-sensititve male sterile rice Peiai 64S. BMC Genomics 16:102. 10.1186/s12864-015-1317-7 25887533 PMC4367915

[B18] Hu R. Xiao L. Bao F. Li X. He Y. (2016). Dehydration-responsive features of *Atrichum undulatum*. J. Plant Res. 129, 945–954. 10.1007/s10265-016-0836-x 27255889 PMC4977332

[B19] Huang H. Møller I. M. Song S. Q. (2012). Proteomics of desiccation tolerance during development and germination of maize embryos. J. Proteomics 75, 1247–1262. 10.1016/j.jprot.2011.10.036 22108046

[B20] Kang Y. J. Kim S. K. Kim M. Y. Lestari P. Kim K. H. Ha B. K.. (2014). Genome sequence of mungbean and insights into evolution within *Vigna* species. Nat. commun. 5, 5443. 10.1038/ncomms6443 25384727 PMC4241982

[B21] Keatinge J. D. H. Easdown W. J. Yang R. Y. Chadha M. L. Shanmugasundaram S. (2011). Overcoming chronic malnutrition in a future warming world: the key importance of mungbean and vegetable soybean. Euphytica 180, 129–141. 10.1007/s10681-011-0401-6

[B22] Kim S. K. Nair R. M. Lee J. Lee S. H. (2015). Genomic resources in mungbean for future breeding programs. Front. Plant Sci. 6:626. 10.3389/fpls.2015.00626 26322067 PMC4530597

[B23] Koster K. L. Leopold A. C. (1988). Sugars and desiccation tolerance in seeds. Plant Physiol. 88, 829–832. 10.1104/pp.88.3.829 16666392 PMC1055669

[B24] Langmead B. Salzberg S. L. (2012). Fast gapped-read alignment with Bowtie 2. Nat. Methods 9, 357–359. 10.1038/nmeth.1923 22388286 PMC3322381

[B25] Ma C. Wang H. Macnish A. J. Estrada-Melo A. C. Lin J. Chang Y. H.. (2015). Transcriptomic analysis reveals numerous diverse protein kinase and transcription factors invoved in desiccation tolearance in the resurrection plant Myrothamnus flabellifolia. Horti. Res. 2:15034. 10.1038/hortres.2015.34 26504577 PMC4595987

[B26] Maia J. Dekkers B. J. Dolle M. J. Ligterink W. Hilhorst H. W. (2014). Abscisic acid (ABA) sensitivity regulates desiccation tolerance in germinated Arabidopsis seeds. New Phytol. 203, 81–93. 10.1111/nph.12785 24697728

[B27] Maia J. Dekkers B. J. W. Provart N. J. Ligterink W. Hihorst HW. (2011). The re- establishment of desiccation tolerance in germinated *Arabidopsis thaliana* seeds and its associated transcriptome. PLoS ONE 6:e29123. 10.1371/journal.pone.0029123 22195004 PMC3237594

[B28] McDonald M. B. (1999). Seed deterioration: physiology, repair and assessment. Seed Sci. Technol. 27, 177–237.

[B29] Michalak M. Barciszewska M. Z. Barciszewski J. Plitta B. P. Chmielarz P. (2013). Global changes in DNA methylation in seeds and seedling of Pyrus communis after seed desiccation and storage. PLoS ONE. 8:e70693. 10.1371/journal.pone.0070693 23940629 PMC3734228

[B30] Mohanty B. Kitazumi A. Cheung C. Y. M. Lakshmanan M. de los Reyes B. G. Jang I. C.. (2016). Indentification of candidate network hubs involved in metabolic adjustments of rice under drought stress by integrating transcriptome data and genomce-scale metabolic network. Plant Sci. 242, 224–239. 10.1016/j.plantsci.2015.09.018 26566840

[B31] Moore J. P. Le N. T. Brandt W. F. Driouich A. Farrant J. M. (2009). Towards a systems-based understanding of plant desiccation tolerance. Trends Plant Sci. 14, 110–117. 10.1016/j.tplants.2008.11.007 19179102

[B32] Pammenter N. W. Berjak P. (1999). A review of recalcitrant seed physiology in relation to desiccation-tolerance mechanisms. Seed Sci. Res. 9, 3–37. 10.1017/S0960258599000033

[B33] Plitta B. P. Michalak M. Bujarska-Borkowska B. Barciszewska M. Z. Barciszewski J. Chmielarz P. (2014). Effect of desiccation on the dynamics of genome-wide DNA methylation in orthodox seeds of *Acer platanoides*. L. Plant Physiol. Biochem. 85, 71–77. 10.1016/j.plaphy.2014.10.014 25394802

[B34] Senaratna T. Mckersie B. D. (1983). Dehydration injury in germinating soybean (*Glycine max* L. Merr) seeds. Plant physiol. 72, 620–624. 10.1104/pp.72.3.620 16663056 PMC1066291

[B35] Shannon P. Markiel A. Ozier O. Baliga N. S. Wang J. T. Ramage D.. (2003). Cytoscape: a software environment for integrated models of biomolecular interaction networks. Genome Res. 13, 2498–2504. 10.1101/gr.1239303 14597658 PMC403769

[B36] Tunnacliffe A. Wise M. J. (2007). The continuing conundrum of the LEA proteins. Naturwissenschaften 94, 791–812. 10.1007/s00114-007-0254-y 17479232

[B37] Verdier J. Lalanne D. Pelletier S. Torres-Jerez I. Righetti K. Bandyopadhyay K. (2013). A regulatory network-base appoach dissects of maturation processes related to ther acquisition of desiccation tolerance and longevity of Medicago truncatula seeds. Plant Physiol. 163, 757–774. 10.1104/pp.113.222380 23929721 PMC3793056

[B38] Vertucci C. W. Farrant J. M. (1995). Acquisition and loss of desiccation tolerance, in Seed Development and Germination, eds Kigel J. Galili G. (New York, NY: Marcel DekkerInc), e237–e271.

[B39] Villalobos M. A. Bartels D. Iturriaga G. (2004). Stress tolerance and glucose insensitive phenotypes in Arabidopsis overexpressing the CpMYB10 transcription factor gene. Plant Physiol. 135, 309–324. 10.1104/pp.103.034199 15122027 PMC429382

[B40] Wang A. H. Hu J. H. Huang X. X. Li X. Zhou G. L. Yan Z. X. (2016). Comparative transcriptome analysis reveals heat-responsive genes in Chinese cabbage (*Brassica rapa* ssp. chinensis). Front. Plant Sci. 7:939. 10.3389/fpls.2016.00939 27443222 PMC4923122

[B41] Wehmeyer N. Vierling E. (2000). Respondes to discrete development signals and suggests a general protective role in desiccation tolerance. Plant Physiol. 122, 1099–1108. 10.1104/pp.122.4.1099 10759505 PMC58944

[B42] Wu J. H. Wang W. Q. Song S. Q. Cheng H. Y. (2009). Reactive oxygen speices scavenging enzymes and down-adjustment of metabolism level in mitochondria associated with desiccation-tolerance acquisition of maiz embryo. J. Integr. Plant Biol. 51, 638–645. 10.1111/j.1744-7909.2009.00841.x 19566642

[B43] Wu X. L. Gong F. P. Yang L. Hu X. L. Tai F. J. Wang W. (2015). Proteomic analysis reveals differential accumulation of small heat shock proteins and late embryogenesis abundant proteins between ABA-deficient mutant vp5 seeds and wild-type Vp5 seeds in maize. Front. Plant Sci. 5:801. 10.3389/fpls.2014.00801 25653661 PMC4299431

[B44] Zemach A. McDaniel I. E. Silva P. Zilberman D. (2010). Genome-wide evolutionary analysis of eukaryotic DNA methylation. Science 328, 916–919. 10.1126/science.1186366 20395474

