# Supplementary figures and images for: Transcriptomic Profiling Reveals Metabolic and Regulatory Pathways in the Desiccation Tolerance of Mungbean (Vigna radiata [L.] R. Wilczek)
Source: Front Plant Sci. 2016 Dec 21;7:1921. doi: 10.3389/fpls.2016.01921 (PMC5174128; doi:10.3389/fpls.2016.01921)

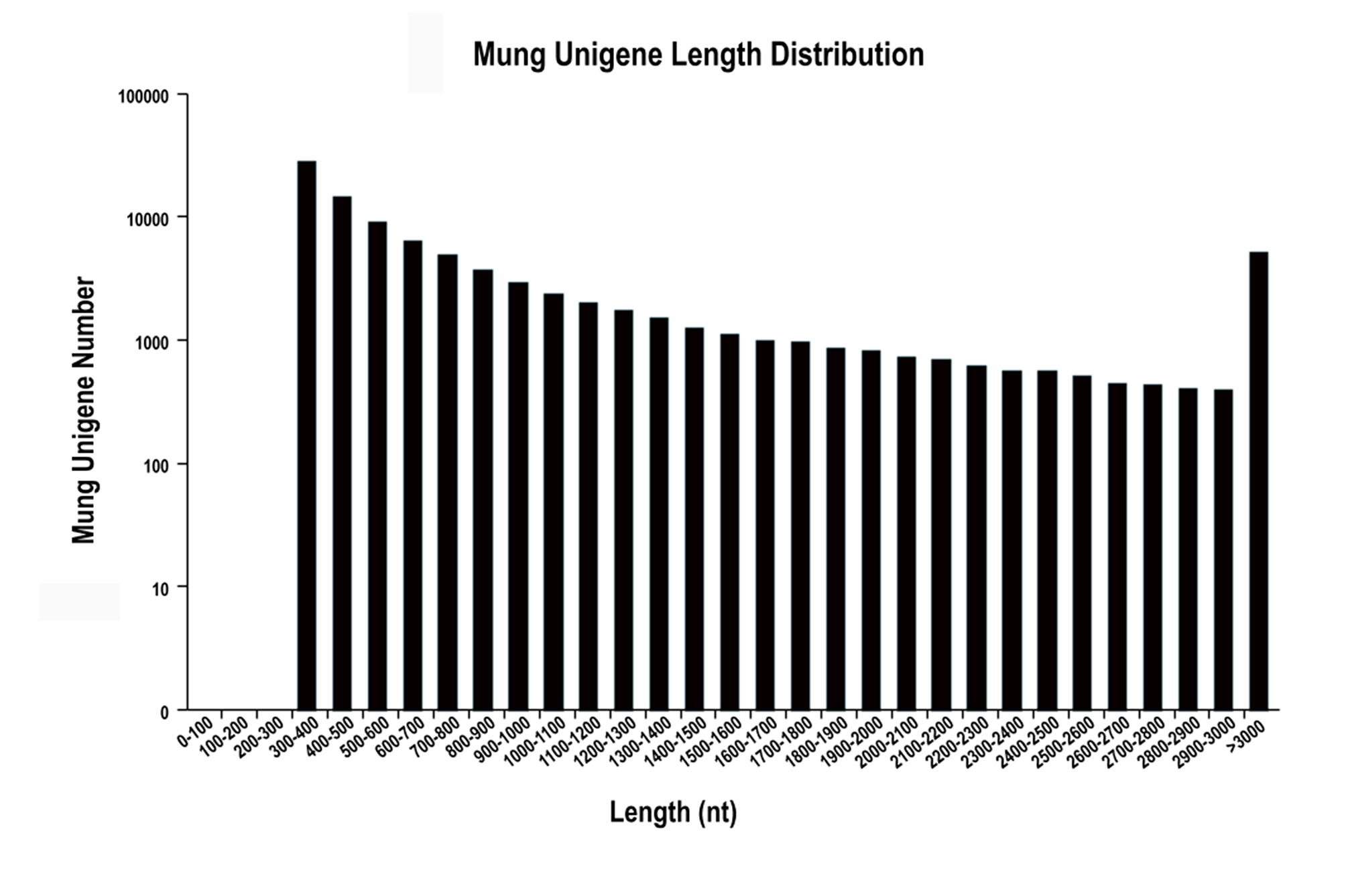

Supplement: Figure S1 — Distribution of the length of transcript assembly of unigenes. [file Image1.TIF]

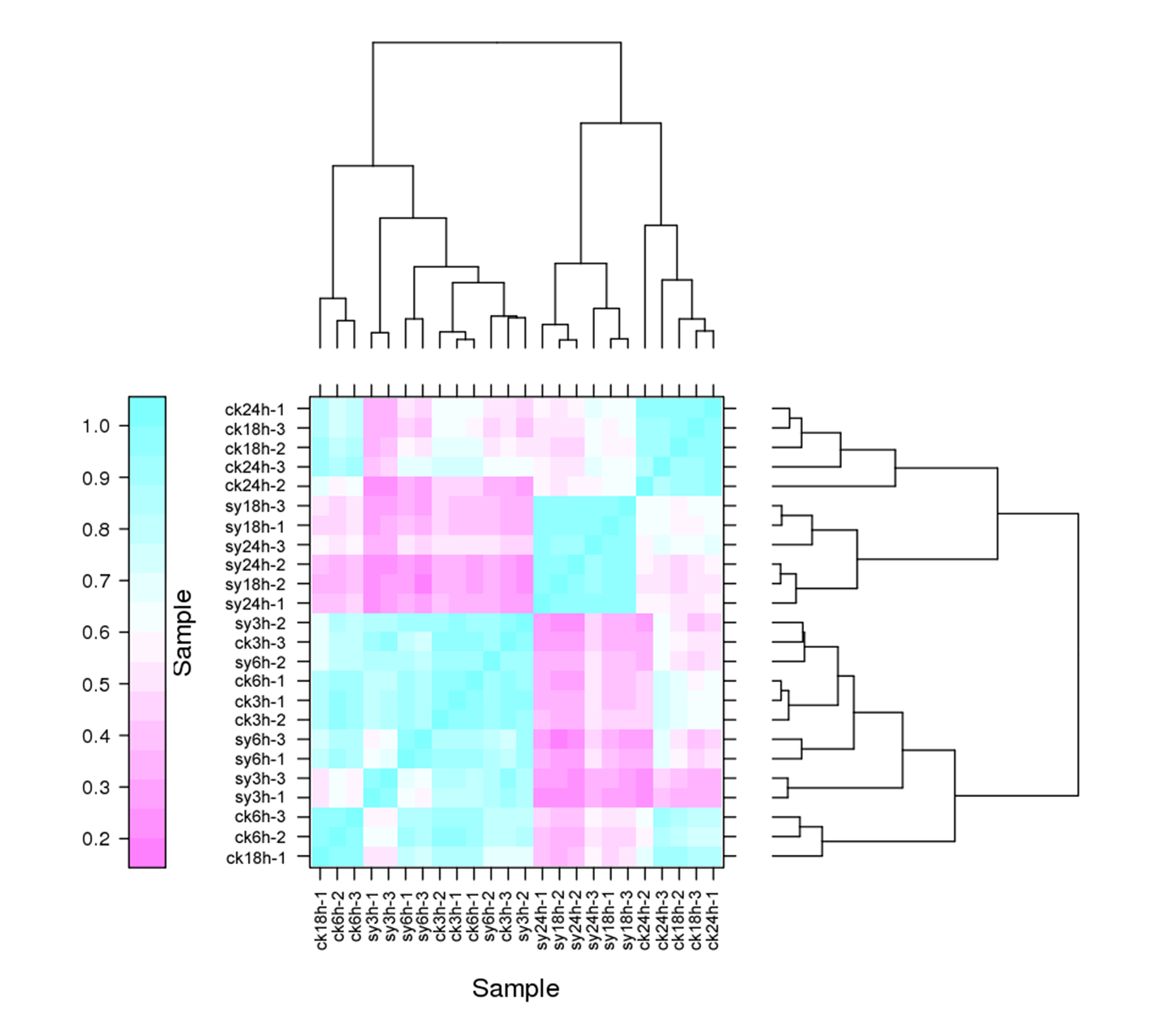

Supplement: Figure S2 — Heatmap of the correlation of the 24 different samples of mungbean. [file Image2.TIFF]

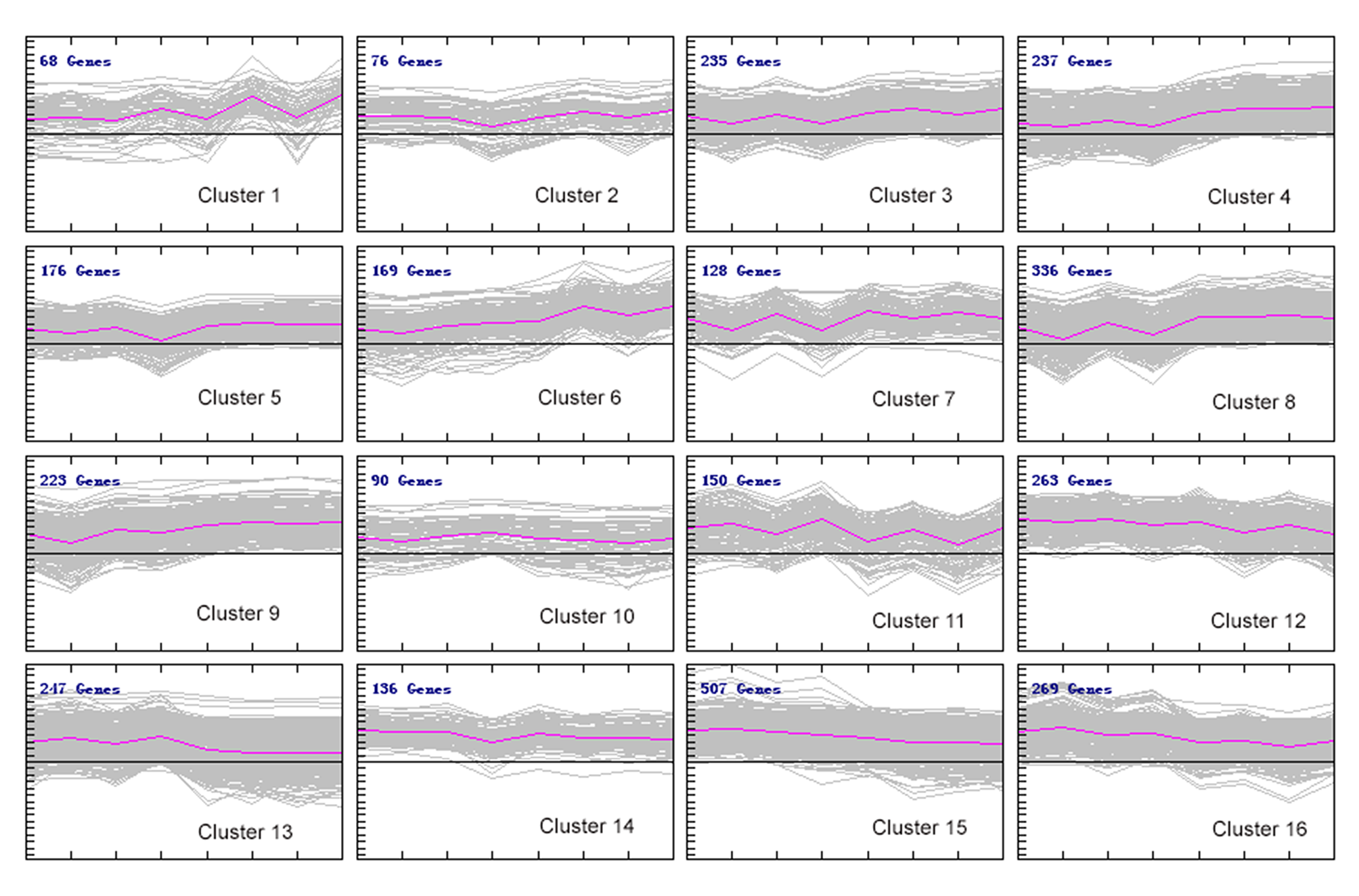

Supplement: Figure S3 — Clustering analysis of the differentially expressed genes (DEGs) at 3–18 h of imbibition. [file Image3.TIFF]

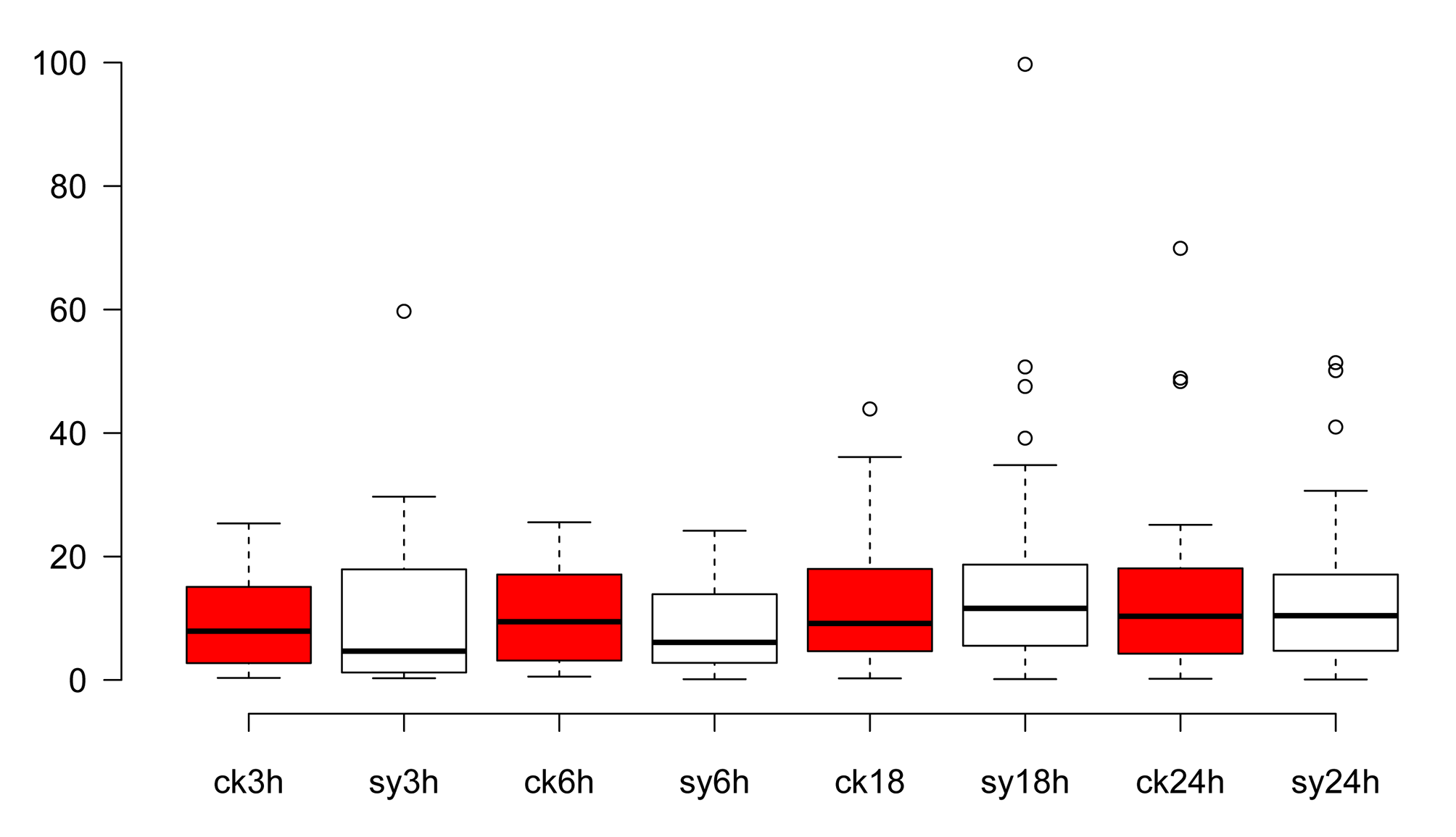

Supplement: Figure S4 — Boxplots of the expression levels of DEGs encoding methyltransferase at different stages in mungbean seeds. [file Image4.TIFF]

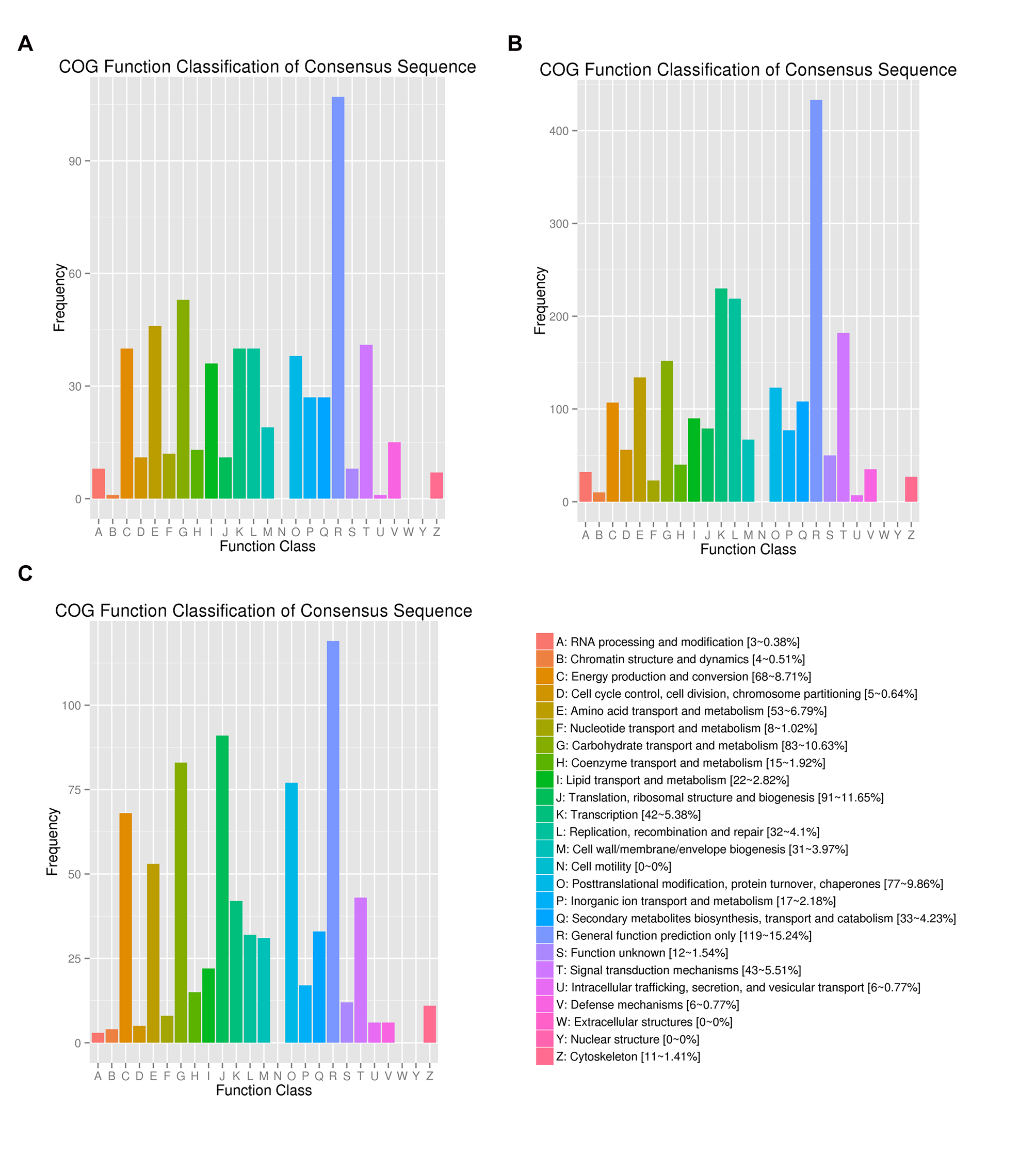

Supplement: Figure S5 — COG functional classification of differentially expressed genes (DEGs) at 3–18 h of imbibition. (A) 3 h, (B) 6 h, and (C) 18 h. [file Image5.TIFF]
